# Treatment options in extra-articular distal radius fractures: a systematic review and meta-analysis

**DOI:** 10.1007/s00068-021-01679-z

**Published:** 2021-05-19

**Authors:** Guido W. Van Oijen, Esther M. M. Van Lieshout, Maarten R. L. Reijnders, Anand Appalsamy, Tjebbe Hagenaars, Michael H. J. Verhofstad

**Affiliations:** Trauma Research Unit, Department of Surgery, Erasmus MC, University Medical Center Rotterdam, P.O. Box 2040, 3000 CA Rotterdam, The Netherlands

**Keywords:** Review, Distal radius fractures, Extra-articular, Treatment, Non-operative, Operative

## Abstract

**Purpose:**

This systematic literature review aimed to make a detailed overview on the clinical and functional outcomes and to get insight into the possible superiority of a treatment method for extra-articular distal radius fractures**.**

**Methods:**

Embase, Medline, Cochrane Library, Web of Science, and Google Scholar were searched for studies describing treatment results. Five treatment modalities were compared: plaster cast immobilization, K-wire fixation, volar plating, external fixation, and intramedullary fixation**.**

**Results:**

Out of 7,054 screened studies, 109 were included in the analysis. Overall complication rate ranged from 9% after plaster cast treatment to 18.5% after K-wire fixation. For radiographic outcomes, only volar tilt in the plaster cast group was lower than in the other groups. Apart from better grip strength after volar plating, no clear functional differences were found across treatment groups.

**Conclusion:**

Current literature does not provide uniform evidence to prove superiority of a particular treatment method when looking at complications, re-interventions, and long-term functional outcomes.

**Supplementary Information:**

The online version contains supplementary material available at 10.1007/s00068-021-01679-z.

## Introduction

A large variety exists in distal radius fractures; from high-energy, comminuted, intra-articular fractures, to low-energy, simple extra-articular fractures. Because of the difference in bone quality, fracture characteristics, associated soft tissue injury, and patient’s needs between these groups, a different treatment approach might be necessary [1–3].

Fractures of the distal radius can be treated operatively or non-operatively. Closed reduction and plaster cast immobilization was traditionally preferred for both intra- and extra-articular fractures. Devices for operative treatment, such as Kirschner wires, plates, external fixators, and intramedullary implants, have been introduced over the last decades and their appropriate use yields good functional results [4–6]. Especially the introduction of volar locking plates has led to a significant increase in operative treatment rates, also for extra-articular fractures, because of the improvement in stability and therefore the possibility for early mobilization [7]. On the other hand, complaints and complications from tendon irritation are assumed more common and the extensive dissection of soft tissue might cause fracture and wound healing problems [7]. Other operative treatments also provide better stability than plaster casting, but have their own cons [8–10].

Current guidelines suggest closed reduction and plaster cast immobilization as the primary treatment for extra-articular distal radius fractures because the treatment is non-invasive and cheap [11, 12]. However, it is unclear if a non-invasive method also means fewer complications and an acceptable loss of function. There is still no consensus regarding the superiority of one of these methods for treating extra-articular distal radius fractures.

The aim of this systematic literature review and meta-analysis was to make a detailed overview on the clinical and functional outcomes and to get insight into the possible superiority of a treatment method in adult patients with an extra-articular distal radius fracture.

## Materials and methods

### Search strategy

A literature search was conducted June 19, 2018. Embase, Medline, The Cochrane Library, Web of Science, and Google Scholar were searched to identify relevant clinical studies that report on the outcomes of extra-articular distal radius fractures. De-duplication of studies was performed as described before [13]. The exact search strategy for the different databases is shown in Online Appendix 1. No language limits were used and any potentially eligible non-English language manuscripts were translated if possible by native speaking colleagues or using Google translate, if no native speaker was available.

### Selection criteria

The studies were eligible for inclusion if they met the following criteria: (1) Patients who suffered and were treated for an extra-articular distal radius fracture; (2) age 18 years or older; (3) patients were either treated using a plaster cast, K-wire(s), plate fixation, external fixator, or an intramedullary device; (4) primary data for at least one outcome parameter had to be available.

Studies without a clear description of fracture type or intervention, or studies that lacked sufficient data for analysis were excluded. In addition, studies with incomplete registration of complications were excluded. Studies describing a not-commonly used device or technique for extra-articular fractures were excluded (*i.e.* dorsal plates, Epibloc system, cannulated screw, above elbow cast, etc.). If a study consisted of one eligible and one non-eligible cohort, this study was included in our study, but only if data for the eligible group was provided.

Two authors (GWVO and AA) independently screened the search results for potentially eligible studies by checking the title and abstract in phase one. Any disagreement has been resolved by consensus or consultation of a third author (TH). After the exclusion of all irrelevant studies, the full text of the remaining studies was obtained, and the eligibility was evaluated to complete selection in phase two. This again was done independently by two authors (GWVO and MRLR). Any disagreement was resolved as described above. If the full-text study was not available, the corresponding author was contacted and asked.

The study protocol has not been registered or published before.

### Data extraction

In phase three, the same two authors independently extracted the following data from the included studies: author, year of publication, study design, type of treatment, number of patients, mean age of patients, number of female patients, duration of follow-up, and type of fracture classification. The outcome data that were retrieved, consisted of; complications and re-interventions, radiographic outcomes (*i.e.* consolidation rate, volar/dorsal tilt, radial inclination, radial height, and ulnar variance), range of motion, grip strength, and functional outcome scores (*i.e.* Disabilities of the Arm, Shoulder, and Hand (DASH) score, *Quick*-DASH score, Patient Rated Wrist Evaluation (PRWE-)score, Visual Analog Scale (VAS) for pain, Gartland and Werley score, and the Mayo Wrist Score). All outcome data were pooled for four different follow-up periods: 0–3 months, > 3–6 months, > 6–12 months, and 12 months or longer (long term).

### Quality assessment

Risk of bias and methodological quality of the included studies were assessed using the Newcastle–Ottawa Quality Assessment Scale. This scale results in a score ranging from 0 to 9. Scores of 8 and 9 were defined as high-quality studies, scores of 5–7 were defined as medium quality, and scores of 1–4 were defined as low quality [14].

### Data analysis

Meta-analysis for binary data was performed using MedCalc for Windows. Heterogeneity testing was performed using Cochran’s Q and the I^2^ statistic. Q is distributed as a chi-square statistic with k (number of studies) minus 1 degrees of freedom. Q has low power as a comprehensive test of heterogeneity [15]. The I^2^ statistic describes the percentage of variation across studies that is due to heterogeneity rather than chance [16, 17]. A random-effects model was used if the I^2^ value was larger than 40%. When the I^2^ was lower than 40% a fixed-effects model was used. Pooled estimates (*i.e.,* proportion) are reported with their 95% confidence intervals (CI). When there was no overlap in the 95% confidence intervals, the difference was considered statistically significant. For the continuous data a pooled estimate (*i.e.,* mean) was calculated in Microsoft Excel, using sample size as a weighting factor. Since most studies only provided the mean values but no standard deviation, 95% confidence interval, or standard error, a formal meta-analysis was not feasible for the continuous data.

## Results

**Fig. 1:**
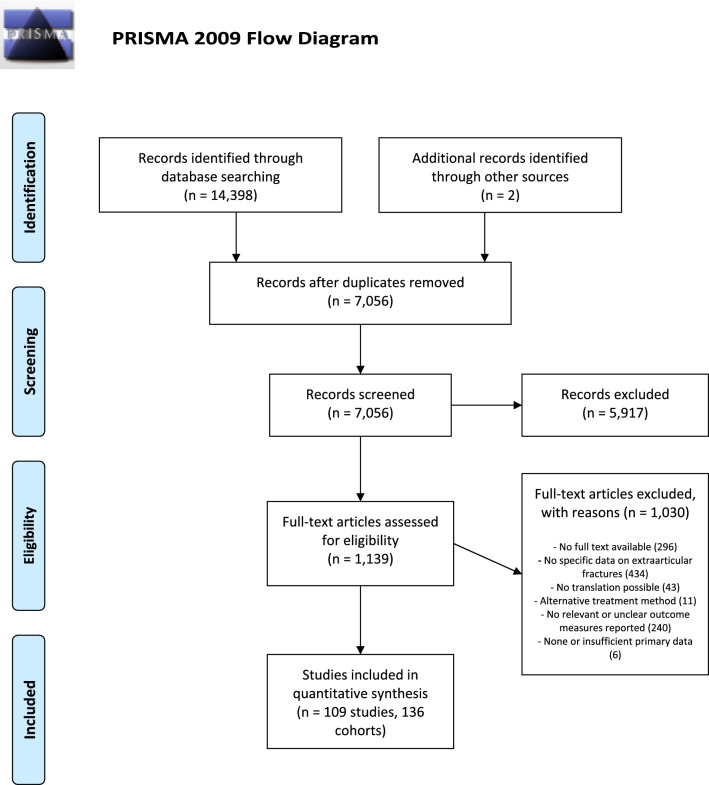
Flowchart of study selection

The primary search resulted in 14,398 hits and after de-duplication 7054 studies remained. After screening these studies by title and abstract 1137 remained for full-text evaluation. A total of 109 studies, with 136 different cohorts were marked eligible for the analysis (Fig. 1 and Supplementary Table S1). These 136 cohorts consisted of a total of 6707 patients divided over the five different treatment modalities. The pooled average age and gender of patients for each treatment method are outlined in Table 1.

**Table 1:** Study subjects per treatment modality

Treatment	Cohorts (*N*)	Population (*N*)	Mean age (95% CI)	Proportion female (95% CI)
Plaster cast	31	2185	61.7 (53.5-69.9)	78.5 (71.9–84.5)
K-wires	37	1525	57.9 (47.2-68.7)	67.2 (56.1–77.4)
Volar plate	41	2245	58.8 (51.7-65.9)	73.2 (63.4–82.0)
External fixator	16	428	50.6 (31.4-69.8)	77.6 (63.4–89.1)
IMN	11	324	59.3 (50.0-68.6)	77.6 (71.3–83.1)

Data are shown as proportion with 95% CI

Among the 109 studies were 21 randomized controlled trials, 37 prospective studies, and 51 retrospective studies (Supplementary Table S1). Among the 136 cohorts, 31 reported on outcomes after plaster cast immobilization [18–48], 37 on K-wire fixation [5, 18, 21, 41, 42, 45, 47, 49–76], 41 on volar plate fixation [4–6, 54–56, 60, 61, 72, 74, 77–107], 16 on external fixation [6, 30, 34, 36, 53, 108–118], and 11 on intramedullary fixation [4, 6, 43, 81, 92, 96, 119–123]. Most of the included studies were of low or medium quality, 40% (*N* = 44) and 50% (*N* = 55), respectively. Ten of the included studies were of high quality, as outlined in Supplementary Table 2.

**Table 2:** Pooled complication rates per treatment modality

Parameter	Treatment	Studies (*N*)	Population (*N*)	Q (*p*-value)	I^2^ (95% CI)	Pooled proportion (95% CI)
Any complication	Plaster cast	9	395	82.8 (< 0.001)	90.3 (84–94)	9.0 (2.0–20.3)
	K-wires	20	644	148.4 (< 0.001)	87.2 (82–91)	18.5 (10.9–27.6)
	Volar plate	21	1,203	63.4 (< 0.001)	68.4 (50–80)	13.3 (9.6–17.4)
	External fixator	11	324	24.0 (0.008)	58.4 (19–79)	18.1 (11.8–25.4)
	IMN	7	198	18.5 (0.005)	67.6 (28–85)	18.2 (9.5–29.0)
CRPS	Plaster cast	9	477	85.4 (< 0.001)	90.6 (84–94)	2.4 (0.0–9.1)
	K-wires	22	878	28.8 (0.119)	27.1 (0–57)	1.1 (0.5–2.0)
	Volar plate	20	842	21.4 (0.317)	11.1 (0–46.4)	2.2 (1.4–3.5)
	External fixator	10	304	12.5 (0.188)	27.9 (0–65.4)	1.4 (0.4–3.4)
	IMN	8	214	5.4 (0.606)	0 (0–58.8)	1.9 (0.5–4.7)
CTS	Plaster cast	7	373	0.4 (0.999)	0 (0–0)	0.4 (0–1.7)*
	K-wires	22	878	8.5 (0.993)	0 (0–0)	0.7 (0.2–1.4)*
	Volar plate	23	941	14.5 (0.884)	0 (0–17)	3.5 (2.4–4.8)*
	External fixator	9	229	0.8 (0.999)	0 (0–0)	0.9 (0.1–3.1)
	IMN	7	198	0.7 (0.994)	0 (0–0)	1.4 (0.3–4.1)
Infection	Plaster cast	10	449	0.8 (1.000)	0 (0–0)	0.5 (0.1–1.7)*
	K-wires	21	707	49.9 (< 0.001)	59.9 (35.3–75.1)	4.7 (2.5–7.6)*
	Volar plate	21	871	7.7 (0.994)	0 (0–0)	0.8 (0.3–1.6)*
	External fixator	12	332	41.8 (< 0.001)	73.7 (53.3–85.2)	10.1 (4.5–17.6)*
	IMN	8	214	0.7 (0.998)	0 (0–0)	0.8 (0.1–3.1)*
Deep infection	Plaster cast	10	449	0.8 (1.000)	0 (0–0)	0.5 (0.1–1.7)
	K-wires	22	839	2.6 (1.000)	0 (0–0)	0.6 (0.2–1.3)
	Volar plate	22	881	3.1 (1.000)	0 (0–0)	0.7 (0.2–1.4)
	External fixator	12	332	4.3 (0.959)	0 (0–0)	0.9 (0.2–2.6)
	IMN	8	214	0.7 (0.998)	0 (0–0)	1.4 (0.3–3.9)
Implant failure	Plaster cast	NA.	NA.	NA.	NA.	NA.
	K-wires	19	587	87.3 (< 0.001)	79.4 (68.5–86.5)	6.1 (2.5–11.1)*
	Volar plate	20	816	17.1 (0.582)	0 (0–42.3)	0.8 (0.3–1.5)*
	External fixator	10	312	5.9 (0.753)	0 (0–42.6)	1.0 (0.2–2.9)
	IMN	8	214	0.7 (0.998)	0 (0–0)	1.4 (0.3–3.9)
Paresthesia superficial radial nerve	Plaster cast	7	339	0.6 (0.997)	0 (0–0)	0.5 (0.0–1.9)*
	K-wires	20	674	17.8 (0.537)	0 (0–44.4)	1.3 (0.6–2.4)*
	Volar plate	18	637	25.2 (0.090)	32.6 (0–61.9)	2.2 (1.0–3.9)
	External fixator	10	305	18.7 (0.028)	52.0 (1.4–76.6)	3.4 (1.0–7.2)
	IMN	8	214	34.3 (< 0.001)	79.6 (60.3–89.5)	10.1 (2.9–21.1)*
Tendon irritation	Plaster cast	9	395	0.8 (0.999)	0 (0–0)	0.5 (0.1–1.8)
	K-wires	19	658	4.6 (0.999)	0 (0–0)	0.7 (0.2–1.7)
	Volar plate	18	775	16.9 (0.461)	0.0 (0–49.7)	2.0 (1.2–3.3)
	External fixator	9	229	0.8 (0.999)	0 (0–0)	0.9 (0.1–3.1)
	IMN	6	182	3.3 (0.651)	0 (0–62.9)	1.0 (0.1–3.7)
Tendon rupture	Plaster cast	8	353	0.8 (0.998)	0 (0–0)	0.5 (0.1–1.9)
	K-wires	20	674	13.0 (0.840)	0 (0–23.9)	1.0 (0.4–2.0)
	Volar plate	20	816	2.6 (1.000)	0 (0–0)	0.8 (0.3–1.7)
	External fixator	9	229	0.8 (0.999)	0 (0–0)	0.9 (0.1–3.1)
	IMN	7	198	6.4 (0.381)	6.1 (0–73.1)	1.2 (0.2–3.9)
Redislocation	Plaster cast	10	500	103.0 (< 0.001)	91.2 (86.1–94.5)	9.3 (2.5–19.9)*
	K-wires	20	763	36.8 (0.008)	48.4 (13.3–69.3)	2.3 (1.0–4.1)
	Volar plate	17	726	12.8 (0.686)	0 (0–39.0)	**1.0** (0.4–2.0)*
	External fixator	10	249	6.3 (0.705)	0 (0–46.9)	2.6 (1.0–5.3)
	IMN	7	198	8.0 (0.236)	25.3 (0–67.34)	2.6 (0.9–5.8)

*Non-overlapping 95% CI.

### Complications

**Fig. 2:**
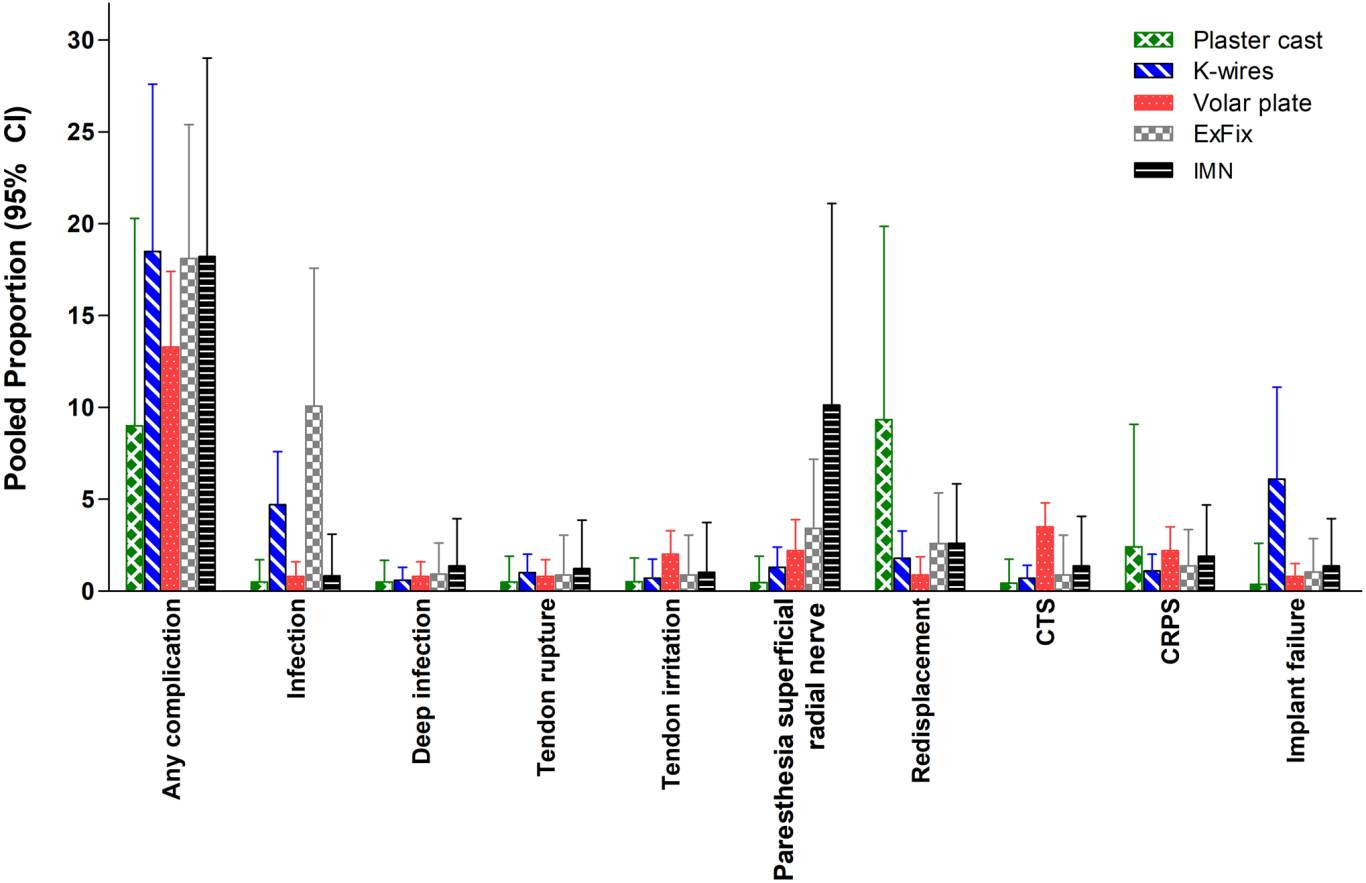
Pooled complication rates per treatment modality. Data are shown as pooled proportion with 95% CI

**Fig. 3:**
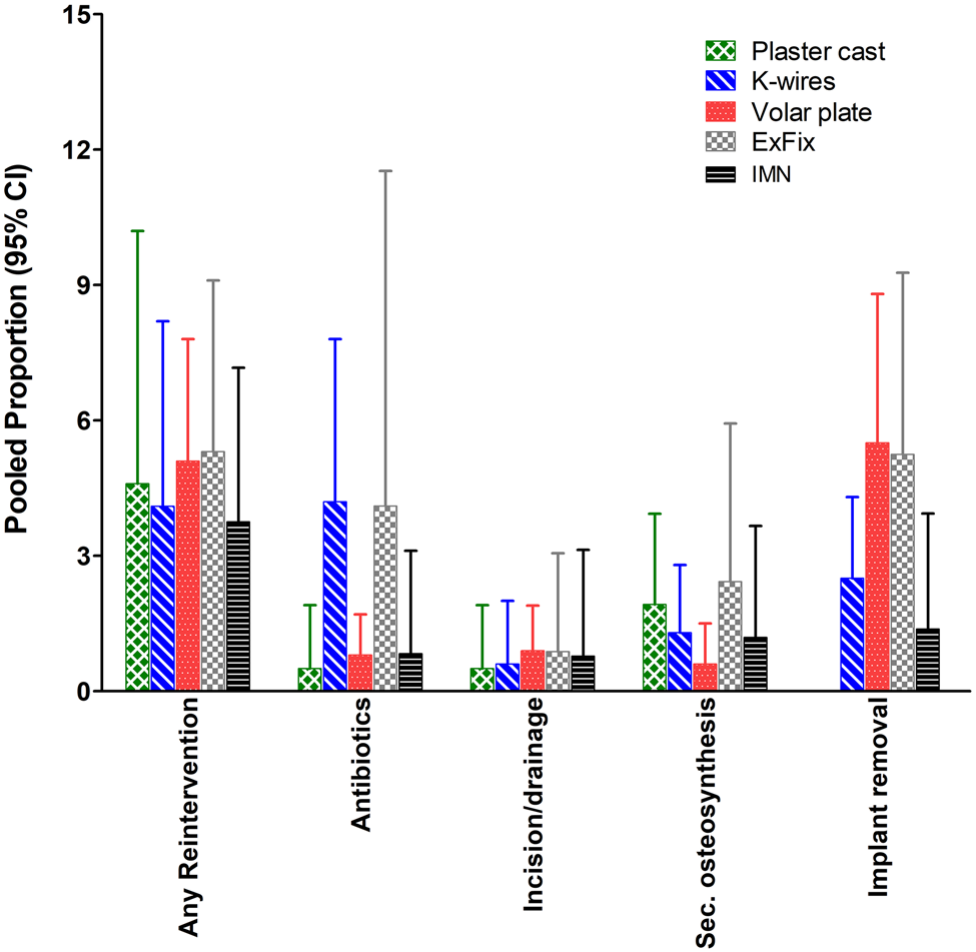
Pooled re-intervention rates per treatment modality. *Early, unplanned removal due to infection, loosening, failure, or other reasons. Data are shown as pooled proportion with 95% CI.

The pooled overall complication rate ranged from 9% after plaster cast treatment to 19% after K-wire fixation (Table 2). Superficial infections were most prominent and higher in the K-wire (4.7% [95% CI 2.5–7.6%]) and ExFix groups (10.1% [95% CI 4.5–17.6%]). Volar plating had the highest rate of carpal tunnel syndrome (3.5% [95% CI 2.4–4.8]) which was higher than plaster cast immobilization and K-wire fixation (0.4 [95% CI 0.0–1.7] and 0.7 [95% CI 0.2–1.4], respectively). In 6.1% of the K-wire group, the implant(s) failed (*i.e.*, loosening or breakage of material). This was significantly higher than in the volar plate fixation group (0.8%). There was significantly less re-dislocation in the volar plate group when compared with plaster cast (1.0% versus 9.3%). Pooled re-intervention rates ranged from 3.8% to 5.3%. Regarding these re-interventions, only one significant difference was found: the use of antibiotics was 4.2% in the K-wire group versus 0.8% in the volar plating group. Data of all other complications and re-interventions had overlapping confidence intervals across the groups and are described in Table 2 and Table 3 and shown in Fig. 2 and Fig. 3.

**Table. 3:** Pooled reintervention rates per treatment modality

Parameter	Treatment	Studies (*N*)	Population (*N*)	Q (*p*-value)	I^2^ (95% CI)	Pooled proportion (95% CI)
Any reintervention	Nonoperative	10	567	57.9 (< 0.001)	84.5 (73.1–91)	4.6 (1.1–10.2)
	K-wires	14	467	45.5 (< 0.001)	71.4 (50.1–83.4)	4.1 (1.4–8.2)
	Volar plate	19	961	46.2 (< 0.001)	61.1 (35.8–76.4)	5.1 (3.0–7.8)
	External fixator	8	217	6.2 (0.521)	0 (0–63.6)	5.3 (2.8–9.1)
	IMN	8	214	11.3 (0.126)	38.1 (0–72.7)	3.8 (1.7–7.2)
Antibiotics	Nonoperative	8	353	0.8 (0.998)	0 (0–0)	0.5 (0.1-1.9)*
	K-wires	15	545	42.5 (< 0.001)	67.0 (43.4-80.8)	4.2 (1.7–7.8)*
	Volar plate	17	757	2.1 (1.000)	0 (0–0)	0.8 (0.3–1.7)*
	External fixator	9	229	36.3 (< 0.001)	78.0 (58.3–88.4)	4.1 (0.4–11.5)
	IMN	8	214	0.7 (0.998)	0 (0–0)	0.8 (0.1–3.1)
Incision and Drainage	Nonoperative	8	353	0.78 (0.998)	0 (0–0)	0.5 (0.1–1.9)
	K-wires	11	393	0.7 (1.000)	0 (0–0)	0.6 (0.1–2.0)
	Volar plate	15	642	2.3 (1.000)	0 (0–0)	0.9 (0.3–1.9)
	External fixator	9	229	0.8 (0.999)	0 (0–0)	0.9 (0.1–3.1)
	IMN	7	202	0.6 (0.997)	0 (0–0)	0.8 (0.1–3.1)
Re-osteosynthesis	Nonoperative	7	353	10.6 (0.157)	34.0 (0–70.8)	1.9 (0.8–3.9)
	K-wires	13	429	9.2 (0.684)	0 (0–43.6)	1.3 (0.4–2.8)
	Volar plate	17	781	6.9 (0.976)	0 (0–0)	0.6 (0.2–1.5)
	External fixator	6	170	2.0 (0.846)	0 (0–39.0)	2.4 (0.7–5.9)
	IMN	8	214	2.4 (0.932)	0 (0–7.6)	1.2 (0.2–3.7)
Non-planned hardware removal	Nonoperative	NA.	NA.	NA.	NA.	NA.
	K-wires	15	497	22.9 (0.062)	38.9 (0–66.9)	2.5 (1.3–4.3)
	Volar plate	18	901	54.5 (< 0.001)	68.8 (49.3–80.8)	5.5 (3.0–8.8)
	External fixator	7	197	5.2 (0.524)	0 (0–66.6)	5.2 (2.6–9.3)
	IMN	8	214	0.7 (0.998)	0 (0–0)	1.4 (0.3–3.9)

*Non-overlapping 95% CI.

### Radiographic outcome

**Fig. 4:**
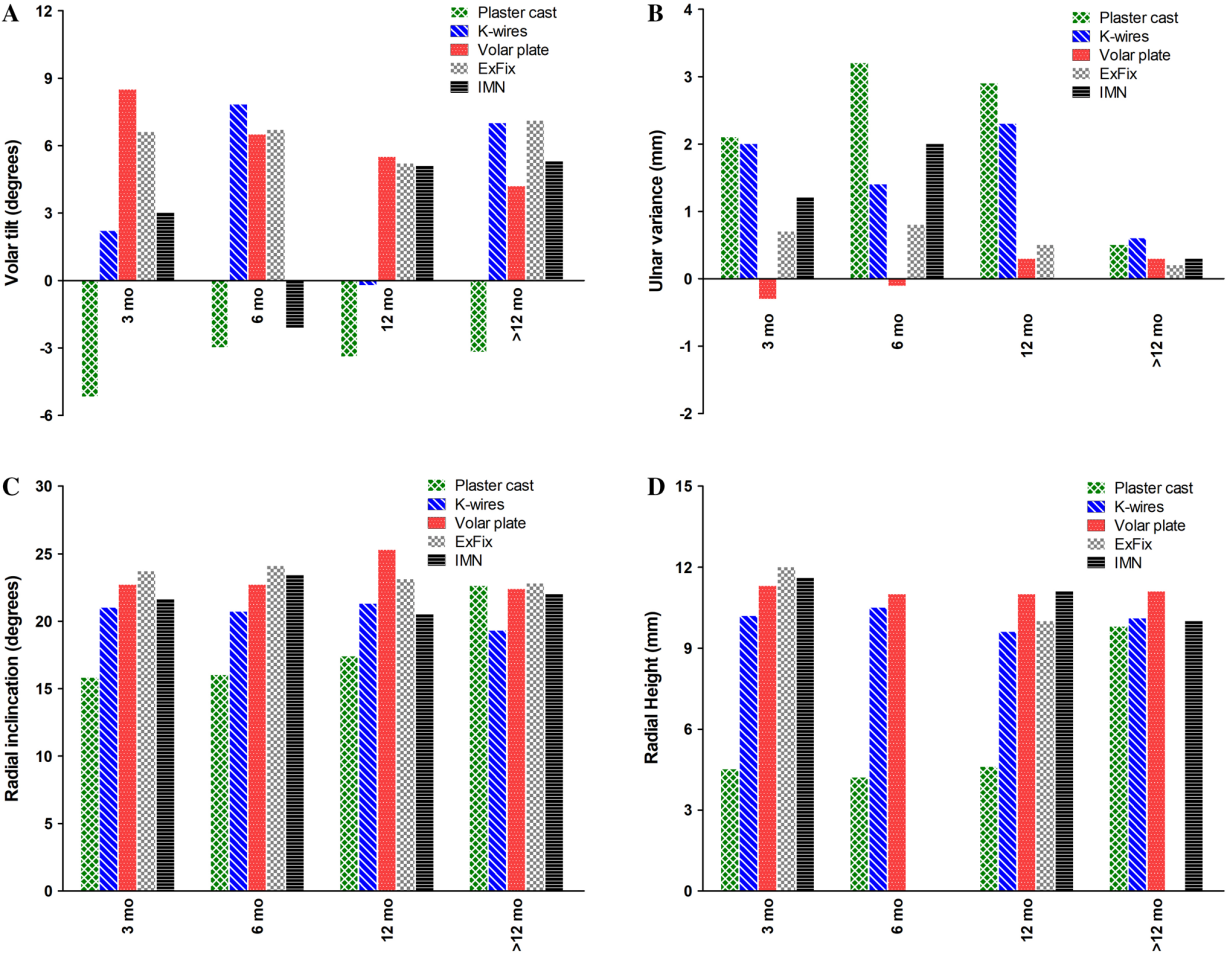
Radiographic outcomes per treatment modality. **a **Volar tilt (degrees), **b** Ulnar variance (mm), **c** Radial inclination (degrees) and **d** Radial height (mm). Data are shown as sample size weighted mean.

Radiographic outcomes are shown in Fig. 4 and Supplemental Table 2. Because no non-union was reported for any of the treatment methods, consolidation rates were not different. The pooled mean radial inclination, radial height, and ulnar variance were similar for all five treatments after > 12 months’ follow-up. Overall, volar plate fixation shows consistent good outcome for all four radiological measurements. Especially the ulnar variance was clearly better than the other four treatment modalities with 0.3 mm, − 0.1 mm, 0.3 mm, and 0.3 mm at 0–3, 3–6, 6–12, and > 12 months after surgery, respectively. The palmar tilt in the plaster cast group was consistently lower than in other groups in all follow-up periods. The proportion of patients with a good or excellent Lidström score was reported for three treatments only: plaster cast immobilization, K-wire fixation and external fixation. Proportions were 72% [95% CI 45.3–92.2], 90% [95% CI 81.3–95.2], and 88% [95% CI 59.9–99.9], respectively.

### Functional clinical outcome

**Fig. 5:**
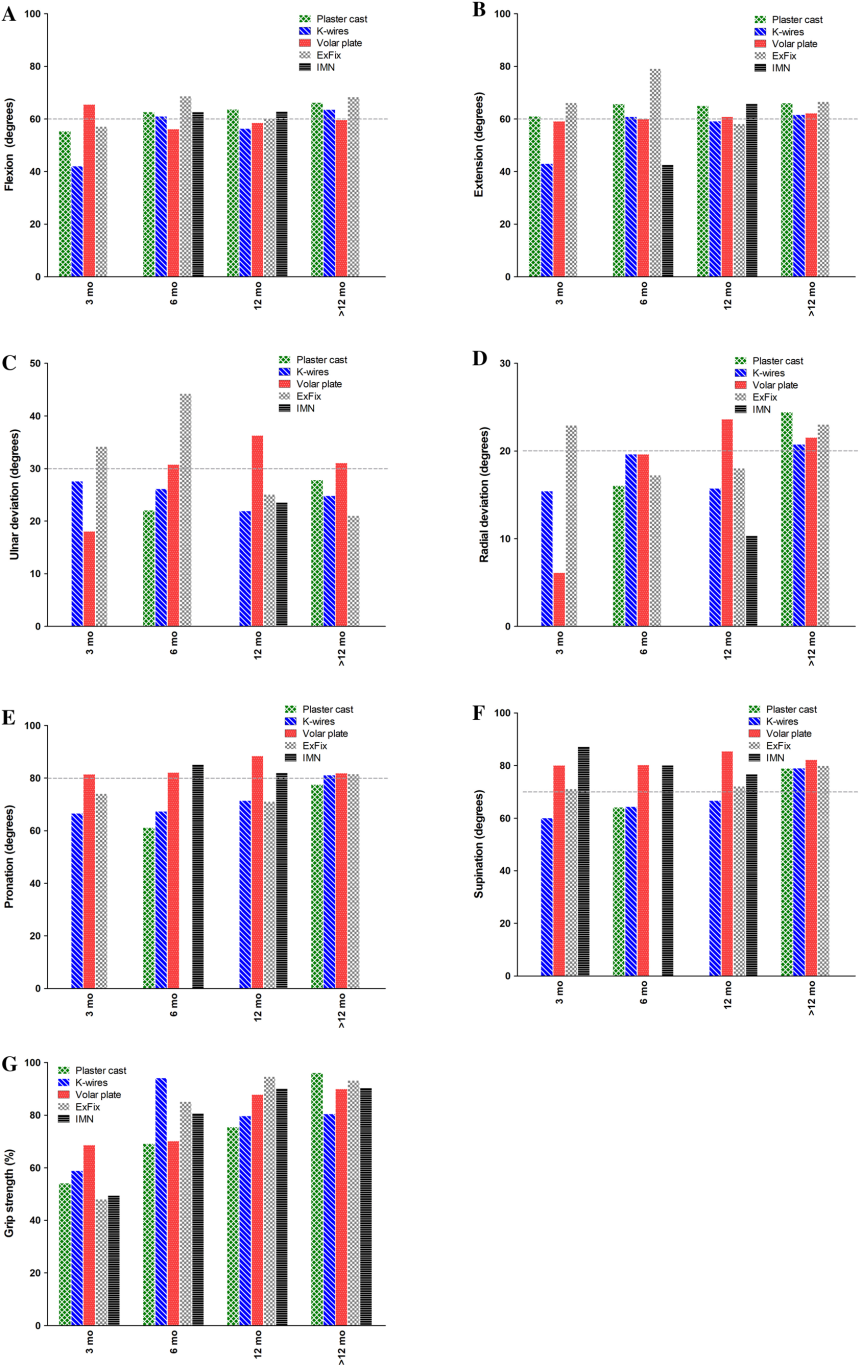
Range of motion and grip strength per treatment modality. **a** Flexion (degrees), **b** Extension (degrees), **c** Ulnar deviation (degrees), **d** Radial deviation (degrees), **e** Pronation (degrees), **f** Supination (degrees), **g** Grip strength (% of contralateral side). Data are shown as sample size weighted mean. The grey line represents the lowest values that will not cause any functional impairment (disability value [127])

Range of motion and grip strength are outlined in Fig. 5 and Supplemental Table 2. For several outcome parameters, none or a small number of studies provided data for analysis. Differences in range of motion and grip strength were especially found in the short-term follow-up. For example, volar plate fixation showed a relatively good grip strength (69% of the non-injured side) and pro-/supination (81.4 and 80.0 degrees, respectively) at 0–3 months’ follow-up. Both pro- and supination were above the limit of disability for the entire follow-up period. At long-term follow-up, no clear functional differences were found across the treatment groups.

### Patient-reported outcomes

**Fig. 6:**
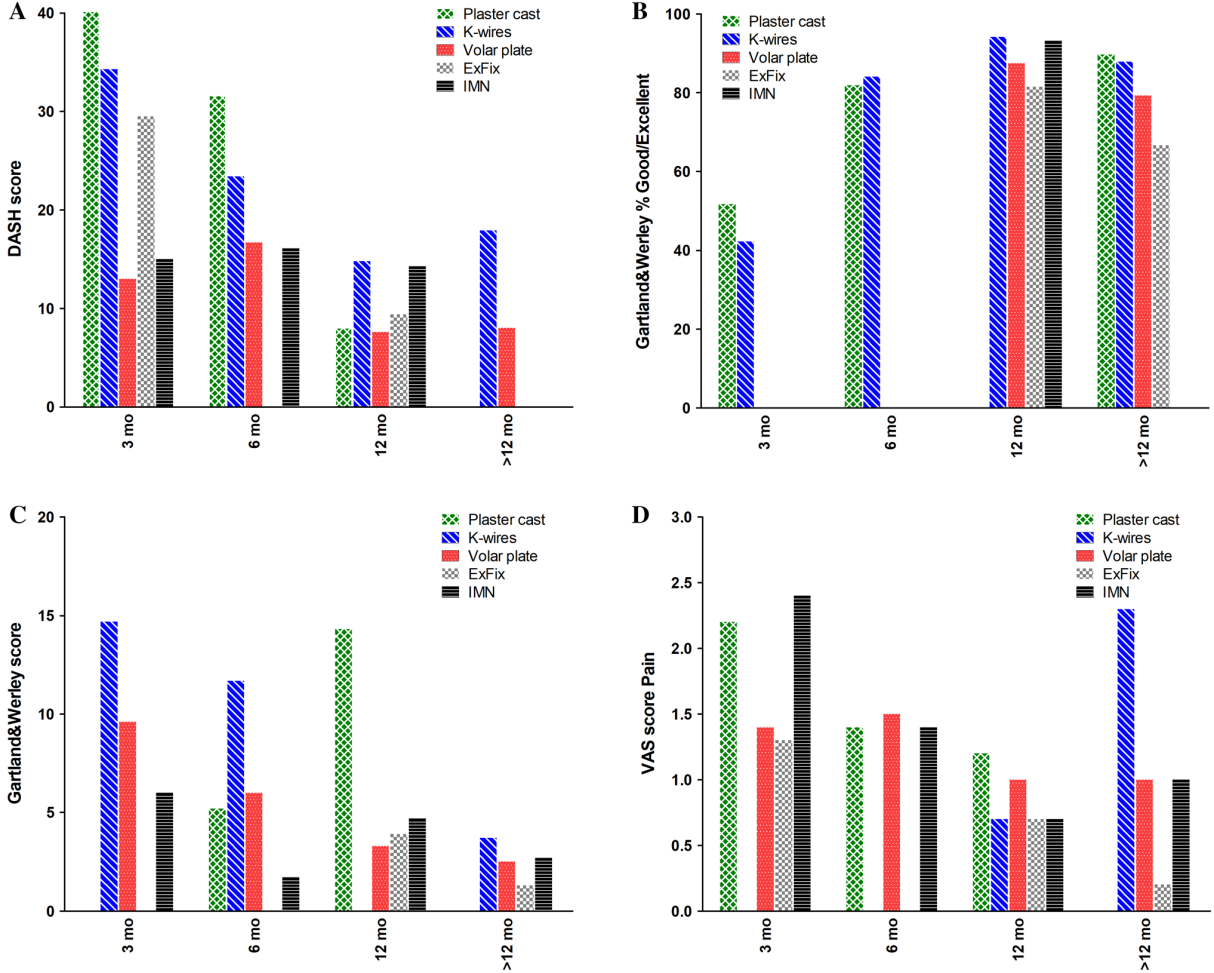
Patient-reported outcome measures per treatment modality. **a** DASH score **b** Gartland & Werley (% good or excellent) **c** Gartland & Werley Score, **d:** VAS score for pain. Data are shown as sample size weighted mean.

A large variety of patient rated outcome measures were used in literature. This resulted in small numbers of study subjects and uncertain values. Three outcome measures that were used most often, were chosen to be analyzed and are shown in Fig. 6: Disabilities of Arm, Shoulder, and Hand (DASH) score, Gartland and Werley score, and a Visual Analog Scale (VAS) for pain. Assessment of the DASH score showed low pooled means for volar plating (13.0 points) and intramedullary fixation (15.0 points) at 0–3 months’ follow-up. Again, at long term, follow-up scores were similar across the treatment groups. For both the Gartland and Werley score and the VAS for pain, an improvement was seen for all treatments over time and no clear differences were found.

## Discussion

This systematic review and meta-analysis aimed to evaluate complications, re-interventions, radiographic outcomes, functional outcomes, and patient-reported outcomes of the most commonly described treatment methods for extra-articular distal radius fractures. The data do not show a clear superiority or inferiority for any of the treatments evaluated.

**Fig. 7:**
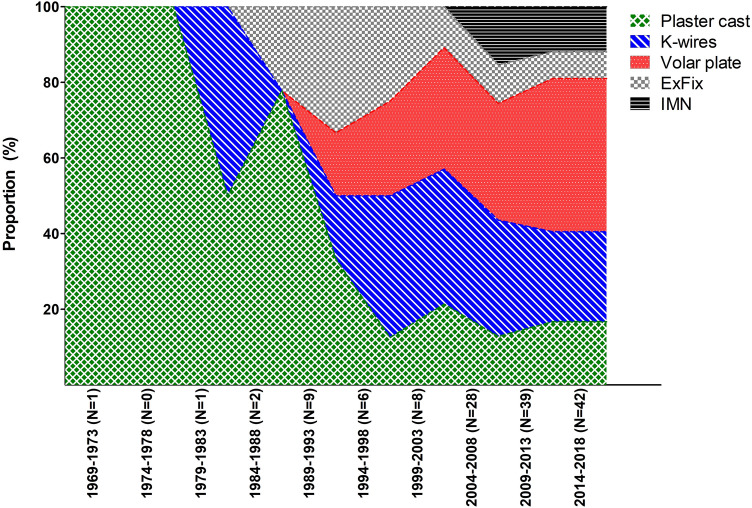
Included studies per treatment modality per time period. Data are shown as proportion of the total included studies per treatment modality in time periods of five years (N = included studies)

The main treatment goals for extra-articular distal radius fractures are to regain an adequate wrist function and/or to release pain. In order to study and objectivize this, a multitude of questionnaires and outcome scores have been introduced over the last decades. However, which outcome parameters should be used for valid and accurate assessment of wrist function are still unclear. Also, the variety of treatment options for distal radius fractures impedes the ability to prove superiority or inferiority of the currently used treatment methods. Cochrane reviews and the American Academy of Orthopaedic Surgeons both labeled the evidence as “inconclusive” [124, 125]; [126]; [12]. This review focused on extra-articular distal radius fractures only, where both Cochrane and the AAOS stated this inconclusiveness almost a decade ago for intra-articular fractures as well. All eligible literature has been included to show trends in techniques used over time. Only volar plates have been subject to significant technological improvement, this probably explains the steep increase of studies in the twenty-first century (Fig. 7).

Surprisingly, unintended outcome has been reported more frequently than intended outcome: 87 out of 136 cohorts in this study reported at least one type of complication. The overall complication and subsequent re-intervention rates are not significantly different for the five treatment modalities. However, not all studies reported consistently on all complications and re-intervention rates. Different definitions of complications and a possible extra focus on specific complications in certain studies, likely induced broad ranges of complication rates, and probably underreporting. For example, finger stiffness was described in only four studies with a rate up to 24% in a plaster cast immobilization cohort of Mardani et al. [18], where the other 105 studies did not even mention it.

No differences in functional and patient-reported outcomes at long-term follow-up (> 12 months) could be detected. This was also mentioned by Costa et al. and Arora et al. [5]; [77]. However, in the short term, differences in functional and patient-reported outcomes seem to exist. In all treatment groups, a lowering trend was observed after the 0–3 months’ follow-up in both the DASH score and the Gartland & Werley score, which means less disability and better function. In line with these findings, also an improvement in grip strength and a decrease in pain were seen in the same time period for all treatment groups. However, volar plates and intramedullary fixation had a much faster recovery than other modalities. Also, range of motion was immediately close to, or even above the limit of disability at 0–3 months’ follow-up for both treatments (Fig. 5). Pronation and supination for all follow-up moments were consistently within the normal range of motion as established by, e.g. the Guides, to the Evaluation of Permanent Impairment by the American Medical Association [127]. This early recovery of patients treated with volar plating and intramedullary fixation might be an important factor in treatment choice. In current society, one might argue that long rehabilitation periods are no longer acceptable for most patients. Moreover, a strong lobby from the industry to sell implants and a high strive to restore anatomy perfectly by surgeons may push towards surgical treatment. These arguments advocate for both short- and long-term cost-effectiveness comparison of the various treatment methods.

Radiological outcomes also seem to be in favor of volar plate fixation, as shown by a positive volar tilt, adequate radial inclination, and a negative or low ulnar variance. Radiographic parameters showed worse outcomes for plaster cast immobilization with concomitant high re-dislocation rates. Diaz-Garcia et al. published a systematic review in 2011 to examine outcomes of unstable distal radius fractures after treatment with either volar locking plate, non-bridging external fixation, bridging external fixation, percutaneous Kirschner-wire fixation, or plaster cast immobilization. They also found that plaster casting was associated with worse radiological outcomes than operative treatment [128]. Our systematic review reveals that since 2011 nothing has really changed.

The first thing that stood out during screening of potential studies, was the small number of studies that specifically reported on different types of distal radius fractures (AO/OTA or other classification systems). Many studies reported treatment outcomes for distal radius fractures in general, without any fracture classification. Distinction between different fracture types is essential to provide adequate treatment. A simple extra-articular fracture requires a different approach than a comminuted intra-articular one.

This systematic review had several limitations. First, most studies that were included were retrospective or prospective observational studies, with disappointing reporting quality for continuous data. For a proper meta-analysis of continuous data the mean, a measure of dispersion (either standard deviation, standard error or confidence interval), and sample size are necessary. Many studies in this review failed to provide these data. Moreover, the number of large, well-designed RCTs on extra-articular fractures is very low, and therefore it is very well possible to have a type 2 error. Second, selection bias in the source studies might have influenced the results of the meta-analysis, since stable and simple extra-articular fractures might have been treated non-operatively, whereas the more dislocated and comminuted fractures might have been treated by volar plating or external fixator. However, current data did not allow for meta-regression analyses. Therefore, it is not entirely certain if the current whether the findings are related to the treatment type, or can be explained by a difference in indication. Third, there was high heterogeneity in type of outcome parameters and time points of measurements reported. Goldhahn et al. mentioned already in 2014 that this heterogeneity is problematic in research on distal radius fractures and suggested a set of core domains to assess outcomes as a possible solution [129]. We have not discovered any clear improvement in data consistency since this article has been published. However, the effect of this study can still be expected over the next years due to its recent character. We agree with Goldhahn et al. that minimal requirements of outcome in the domains patient history, physical examination and radiological findings should be established and journals should only publish papers that meet these criteria. Only then the value of future systematic reviews in the field of orthopedic trauma will increase.

## Conclusion

Current literature does not provide enough evidence to support superiority of a particular treatment method for extra-articular distal radius fractures, when looking at complications, re-interventions, and long-term functional outcomes. A broad range of outcome parameters have been used, which makes the data partly impossible to compare. From a methodological point of view, the quality of data used in this systematic review and subsequent conclusions that can be drawn, appear to be rather weak. This paper should therefore encourage future investigators to use more sound research methods. Consensus on outcome measures and completeness of reporting is necessary to conduct high-quality studies with standardized outcome assessment. This is needed to be able to draw sound conclusions on superiority of one of the treatment methods.

## Supplementary Information

Below is the link to the electronic supplementary material.

Supplementary file1 (DOC 63 kb)

Supplementary file2 (DOCX 17 kb)

Supplementary file3 (DOCX 190 kb)

Supplementary file4 (DOCX 28 kb)

Supplementary file5 (DOCX 24 kb)

Supplementary file6 (DOCX 23 kb)

Supplementary file7 (DOCX 24 kb)

Supplementary file8 (DOCX 24 kb)

## Data Availability

Not applicable.
