# Supplementary material for: Treatment options in extra-articular distal radius fractures: a systematic review and meta-analysis
Source: Eur J Trauma Emerg Surg. 2021 May 19;48(6):4333–48. doi: 10.1007/s00068-021-01679-z (PMC9712287; doi:10.1007/s00068-021-01679-z)
Supplement: Supplementary file 2 — Supplementary file2 (DOCX 17 kb) [file 68_2021_1679_MOESM2_ESM.docx]

**APPENDIX 1. Search strategy. June 19, 2018**

**Embase.com: 5224 unique studies**

('radius fracture'/exp OR 'Colles fracture'/exp OR (((radius OR radial OR radii OR colles OR smith* ) NEAR/10 (fracture* OR fixat* OR plate OR wire* OR pin OR pins OR nail*))):ab,ti) AND (wrist/de OR 'wrist injury'/de OR 'wrist fracture'/de OR 'Colles fracture'/exp OR (wrist* OR distal* OR (low* NEXT/1 end) OR ((colles OR smith*) NEAR/10 (fracture* OR type))):ab,ti) AND (therapy/exp OR therapy:lnk OR surgery/exp OR surgery:lnk OR 'bone plate'/exp OR 'surgical wire'/exp OR 'orthopedic fixation device'/exp OR 'orthopedic cast'/exp OR 'clinical trial'/exp OR 'treatment outcome'/exp OR (therap* OR treat* OR outcome* OR surg* OR operati* OR nonsurg* OR nonoperati* OR fixat* OR plate OR plating OR wire OR wires OR snare OR snares OR rod OR rods OR Kirschner OR pin OR pins OR pen OR pens OR nail OR nails OR nailing OR interven* OR conservativ* OR cast OR casting OR plaster* OR intramedull* OR endomedull* OR medull* OR kapandji* OR percutan* OR ((close* OR open OR fracture*) NEAR/3 reduc*) OR reduction* OR reposition* OR stabili* OR trial* OR manage*):ab,ti) AND ('clinical study'/de OR 'case control study'/exp OR 'clinical article'/de OR 'clinical trial'/exp OR 'cohort analysis'/exp OR 'follow up'/exp OR 'intervention study'/exp OR 'longitudinal study'/exp OR 'major clinical study'/exp OR 'prospective study'/exp OR 'retrospective study'/exp OR 'controlled study'/exp OR 'clinical evaluation'/exp OR 'evaluation study'/exp OR 'comparative study'/exp OR (trial* OR clinical* OR random* OR cohort* OR longitudinal* OR retrospectiv* OR prospectiv* OR (follow* NEXT/1 up*) OR followup* OR (intervention* NEAR/3 stud*) OR (control* NEAR/3 (stud* OR group*)) OR (case* NEAR/3 serie*) OR patients OR cases OR evaluat* OR comparative*):ab,ti) NOT ((child/exp OR pediatrics/exp OR (child* OR infan* OR pediatr* OR paediatr*):ab,ti) NOT (adult/exp OR (adult* OR elderl*):ab,ti)) NOT ([animals]/lim NOT [humans]/lim) NOT ([Conference Abstract]/lim OR [Letter]/lim OR [Note]/lim OR [Editorial]/lim)

**Medline Ovid: 804 unique studies**

(exp "Radius Fractures"/ OR (((radius OR radial OR radii OR colles OR smith* ) ADJ10 (fracture* OR fixat* OR plate OR wire* OR pin OR pins OR nail*))).ab,ti.) AND (wrist/ OR "wrist injuries"/ OR "Colles' Fracture"/ OR (wrist* OR distal* OR (low* ADJ end) OR ((colles OR smith*) ADJ10 (fracture* OR type))).ab,ti.) AND (exp therapeutics/ OR therapy.xs. OR exp "Surgical Procedures, Operative"/ OR surgery.xs. OR exp "Orthopedic Fixation Devices"/ OR exp "clinical trial"/ OR exp "treatment outcome"/ OR (therap* OR treat* OR outcome* OR surg* OR operati* OR nonsurg* OR nonoperati* OR fixat* OR plate OR plating OR wire OR wires OR snare OR snares OR rod OR rods OR Kirschner OR pin OR pins OR pen OR pens OR nail OR nails OR nailing OR interven* OR conservativ* OR cast OR casting OR plaster* OR intramedull* OR endomedull* OR medull* OR kapandji* OR percutan* OR ((close* OR open OR fracture*) ADJ3 reduc*) OR reduction* OR reposition* OR stabili* OR trial* OR manage*).ab,ti.) AND ("case control study"/ OR exp "clinical trial"/ OR exp "cohort studies"/ OR "Intervention Studies"/ OR OR OR "evaluation studies"/ OR "comparative study"/ OR (trial* OR clinical* OR random* OR cohort* OR longitudinal* OR retrospectiv* OR prospectiv* OR (follow* ADJ up*) OR followup* OR (intervention* ADJ3 stud*) OR (control* ADJ3 (stud* OR group*)) OR (case* ADJ3 serie*) OR patients OR cases OR evaluat* OR comparative*).ab,ti.) NOT ((exp child/ OR exp infant/ OR exp Pediatrics/ OR (child* OR infan* OR pediatr* OR paediatr*).ab,ti.) NOT (exp adult/ OR (adult* OR elderl*).ab,ti.)) NOT (exp animals/ NOT humans/) NOT (letter OR news OR comment OR editorial OR congresses OR abstracts).pt.

**Cochrane: 292 unique studies**

((((radius OR radial OR radii OR colles OR smith* ) NEAR/10 (fracture* OR fixat* OR plate OR wire* OR pin OR pins OR nail*))):ab,ti) AND ((wrist* OR distal* OR (low* NEXT/1 end) OR ((colles OR smith*) NEAR/10 (fracture* OR type))):ab,ti) AND ((therap* OR treat* OR outcome* OR surg* OR operati* OR nonsurg* OR nonoperati* OR fixat* OR plate OR plating OR wire OR wires OR snare OR snares OR rod OR rods OR Kirschner OR pin OR pins OR pen OR pens OR nail OR nails OR nailing OR interven* OR conservativ* OR cast OR casting OR plaster* OR intramedull* OR endomedull* OR medull* OR kapandji* OR percutan* OR ((close* OR open OR fracture*) NEAR/3 reduc*) OR reduction* OR reposition* OR stabili* OR trial* OR manage*):ab,ti)

**Web-of-science: 698 unique studies**

TS=(((((radius OR radial OR radii OR colles OR smith* ) NEAR/10 (fracture* OR fixat* OR plate OR wire* OR pin OR pins OR nail*)))) AND ((wrist* OR distal* OR (low* NEAR/1 end) OR ((colles OR smith*) NEAR/10 (fracture* OR type)))) AND ((therap* OR treat* OR outcome* OR surg* OR operati* OR nonsurg* OR nonoperati* OR fixat* OR plate OR plating OR wire OR wires OR snare OR snares OR rod OR rods OR Kirschner OR pin OR pins OR pen OR pens OR nail OR nails OR nailing OR interven* OR conservativ* OR cast OR casting OR plaster* OR intramedull* OR endomedull* OR medull* OR kapandji* OR percutan* OR ((close* OR open OR fracture*) NEAR/2 reduc*) OR reduction* OR reposition* OR stabili* OR trial* OR manage*)) AND ((trial* OR clinical* OR random* OR cohort* OR longitudinal* OR retrospectiv* OR prospectiv* OR (follow* NEAR/1 up) OR followup* OR (intervention* NEAR/2 stud*) OR (control* NEAR/2 (stud* OR group*)) OR (case* NEAR/2 serie*) OR patients OR cases OR evaluat* OR comparative*)) NOT (((child* OR infan* OR pediatr* OR paediatr*)) NOT ((adult* OR elderl*))) ) AND DT=(article)

**Google scholar: 36 unique studies**

"distal radius|radial fracture|fractures"|"fracture|fractures ** distal radius"|"colles|smith fracture" therapy|treatment|outcome|surgery|operative|nonsurgical|nonoperative|fixation|conservative|cast|casting|plaster
