# Supplementary material for: Treatment options in extra-articular distal radius fractures: a systematic review and meta-analysis
Source: Eur J Trauma Emerg Surg. 2021 May 19;48(6):4333–48. doi: 10.1007/s00068-021-01679-z (PMC9712287; doi:10.1007/s00068-021-01679-z)
Supplement: Supplementary file 3 — Supplementary file3 (DOCX 190 kb) [file 68_2021_1679_MOESM3_ESM.docx]

**Supplementary Table 1: Included cohorts, sorted by treatment.**

| **Publication** | **Year** | **Treatment** | **N (subjects)** | **Follow-up (months)** | **Study design** |
| --- | --- | --- | --- | --- | --- |
| Gutiérrez-Monclus *et al.[47]* | 2018 | 1 | 180 | 6 | Prospective case series |
| Gutiérrez-Espinoza *et al.*[28] | 2017 | 1 | 74 | 6 | RCT |
| Venkatesh *et al.*[44] | 2016 | 1 | 30 | 6 | RCT |
| Baruah *et al.*[21] | 2015 | 1 | 54 | 5.5 | Prospective case series |
| Kumaravel *et al.*[33] | 2015 | 1 | 25 | 12 | RCT |
| Subramaniam *et al.*[41] | 2015 | 1 | 10 | 12 | RCT |
| Jellad *et al.*[30] | 2014 | 1 | 90 | 9 | Prospective cohort |
| Bentohami *et al.*[23] | 2013 | 1 | 257 | 36 | Retrospective case series |
| Tan *et al.*[42] | 2012 | 1 | 19 | 12 | Retrospective cohort |
| Eastley *et al.*[25] | 2012 | 1 | 138 | N.D. | Retrospective cohort |
| Mardani *et al.*[17] | 2011 | 1 | 99 | 3 | RCT |
| Wong, *et al.*[46] | 2010 | 1 | 30 | 19 | RCT |
| Kumar *et al.*[32] | 2008 | 1 | 96 | 18 | Retrospective cohort |
| Rajan *et al.*[36] | 2008 | 1 | 64 | 6 | Prospective cohort |
| Kulej *et al.*[31] | 2007 | 1 | 33 | 20.5 | Prospective case series |
| Azzopardi *et al.*[20] | 2005 | 1 | 27 | 12 | RCT |
| Moroni *et al.*[35] | 2004 | 1 | 20 | 3 | RCT |
| Leone *et al.*[34] | 2004 | 1 | 71 | 6 | Retrospective case series |
| Smilovic *et al.*[37] | 2003 | 1 | 54 | 6 | Prospective case series |
| Stoffelen *et al.*[40] | 1998 | 1 | 50 | 12 | RCT |
| Basso *et al.*[22] | 1998 | 1 | 38 | 2 | Prospective cohort |
| Tsukazaki *et al.*[43] | 1993 | 1 | 58 | 55.2 | Retrospective case series |
| Field *et al.*[26] | 1992 | 1 | 29 | 120 | Retrospective case series |
| Gupta *et al.*[27] | 1991 | 1 | 147 | 15 | Prospective cohort |
| Stein *et al.*[38] | 1990 | 1 | 64 | 6 | Prospective cohort |
| Horne *et al.*[29] | 1990 | 1 | 14 | 15 | RCT |
| Af Ekenstam *et al.*[19] | 1989 | 1 | 22 | 24 | RCT |
| Abbaszadegan *et al.*[18] | 1989 | 1 | 135 | 2 | Prospective case series |
| Stewart *et al.*[39] | 1985 | 1 | 162 | 6 | Prospective cohort |
| Wahlstrom *et al.*[45] | 1982 | 1 | 42 | 1 | Prospective cohort |
| Blitchert *et al.*[24] | 1971 | 1 | 53 | 18 | Prospective cohort |
|  |  |  |  |  |  |
| Camus *et al.[75]* | 2018 | 2 | 17 | 21 | Retrospective cohort |
| Manrique *et al.[73]* | 2017 | 2 | 36 | 12 | RCT |
| Vasudevan *et al.[74]* | 2017 | 2 | 176 | 36 | Retrospective cohort |
| Panthi *et al.*[62] | 2017 | 2 | 90 | 6 | Prospective case series |
| Venkatesh *et al.*[44] | 2016 | 2 | 30 | 6 | RCT |
| Chuang *et al.*[49] | 2015 | 2 | 85 | 3 | Retrospective case series |
| Subramaniam *et al.*[41] | 2015 | 2 | 9 | 12 | RCT |
| Costa *et al.*[8] | 2015 | 2 | 110 | 12 | RCT |
| Yang *et al.*[72] | 2014 | 2 | 25 | 3 | Retrospective cohort |
| Yang *et al.*[72] | 2014 | 2 | 60 | 3 | Retrospective cohort |
| Maire *et al.*[59] | 2013 | 2 | 13 | 40 | Prospective cohort |
| Mirhamidi *et al.*[61] | 2013 | 2 | 23 | 6 | RCT |
| Mirhamidi *et al.*[61] | 2013 | 2 | 22 | 6 | RCT |
| Schneiders *et al.*[67] | 2012 | 2 | 37 | 67.2 | Retrospective cohort |
| Mardani *et al.*[17] | 2011 | 2 | 99 | 3 | RCT |
| Hull *et al.*[54] | 2011 | 2 | 17 | N.D. | Retrospective cohort |
| McFayden *et al.*[60] | 2011 | 2 | 29 | 6 | RCT |
| Das *et al.*[51] | 2011 | 2 | 32 | 6 | Prospective cohort |
| Wong *et al.*[46] | 2010 | 2 | 30 | 20 | RCT |
| Huard *et al.*[53] | 2010 | 2 | 15 | 22 | Retrospective cohort |
| Kennedy *et al.*[56] | 2010 | 2 | 71 | 1.5 | Retrospective case series |
| Sadighi *et al.*[66] | 2010 | 2 | 50 | 3 | Prospective case series |
| Kurup *et al.*[58] | 2008 | 2 | 28 | 1 | Retrospective case series |
| van Aaken *et al.*[69] | 2008 | 2 | 15 | 30 | Retrospective case series |
| Vatansever *et al.*[70] | 2007 | 2 | 42 | 7.86 | Prospective cohort |
| Szyluk *et al.*[68] | 2007 | 2 | 52 | 10 | Retrospective case series |
| Voigt *et al.*[71] | 2006 | 2 | 14 | 26 | Retrospective cohort |
| Rosati, *et al.*[63] | 2006 | 2 | 46 | 56.7 | Retrospective case series |
| Jubel *et al.*[55] | 2005 | 2 | 29 | 29 | Retrospective cohort |
| Azzopardi *et al.*[20] | 2005 | 2 | 27 | 12 | RCT |
| Ruschel *et al.*[65] | 2005 | 2 | 29 | 12 | Prospective case series |
| Kurup *et al.*[57] | 2005 | 2 | 55 | N.D. | Retrospective case series |
| Rosenthal *et al.*[64] | 2002 | 2 | 18 | 2.6 | Prospective case series |
| Franck *et al.*[52] | 2000 | 2 | 20 | 6 | RCT |
| Brady *et al.*[48] | 1999 | 2 | 11 | 11.5 | Retrospective case series |
| Stoffelen *et al.*[40] | 1998 | 2 | 48 | 12 | RCT |
| Clancey *et al.*[50] | 1984 | 2 | 15 | 12 | Prospective case series |
|  |  |  |  |  |  |
| Shimura *et al.[106]* | 2018 | 3 | 10 | 39.1 | Retrospective cohort |
| Selles *et al.[105]* | 2018 | 3 | 125 | 12 | Retrospective cohort |
| Manrique *et al.[73]* | 2017 | 3 | 31 | 12 | RCT |
| Thorninger *et al.*[101] | 2017 | 3 | 374 | 38.4 | Retrospective case series |
| Zhang *et al.*[103] | 2017 | 3 | 98 | 24 | Prospective cohort |
| Naito *et al.*[89] | 2016 | 3 | 8 | 3 | Retrospective case series |
| Solarino *et al.*[95] | 2016 | 3 | 17 | 12 | Retrospective cohort |
| Disseldorp *et al.*[81] | 2016 | 3 | 21 | 50 | Retrospective cohort |
| Plate *et al.*[91] | 2015 | 3 | 30 | 24 | RCT |
| Yamashita *et al.*[102] | 2015 | 3 | 106 | 11 | Retrospective cohort |
| Huffaker *et al.*[85] | 2015 | 3 | 158 | 4.2 | Retrospective case series |
| Häberle *et al.*[84] | 2015 | 3 | 60 | 3 | RCT |
| Costa *et al.*[8] | 2015 | 3 | 105 | 12 | RCT |
| Gereli *et al.*[82] | 2014 | 3 | 31 | 35 | Retrospective cohort |
| Gradl *et al.*[7] | 2014 | 3 | 55 | 24 | RCT |
| Lebailly *et al.*[87] | 2014 | 3 | 83 | 4.1 | Retrospective case series |
| Aita *et al.*[9] | 2014 | 3 | 16 | 12 | RCT |
| Maire *et al.*[59] | 2013 | 3 | 15 | 40 | Prospective cohort |
| Mignemi *et al.*[88] | 2013 | 3 | 37 | 3 | Retrospective case series |
| Braziulis *et al.*[79] | 2013 | 3 | 28 | 6 | Retrospective cohort |
| Hull *et al.*[54] | 2011 | 3 | 17 | N.D. | Retrospective cohort |
| Chappuis *et al.*[80] | 2011 | 3 | 15 | 6 | RCT |
| McFayden *et al.*[60] | 2011 | 3 | 3 | 6 | RCT |
| Geyer *et al.*[83] | 2011 | 3 | 20 | 4 | Prospective case series |
| Souer, *et al.*[97] | 2011 | 3 | 74 | 24 | Retrospective case control |
| Souer, *et al.*[98] | 2010 | 3 | 62 | 24 | Retrospective cohort |
| Huard *et al.*[53] | 2010 | 3 | 10 | 16 | Retrospective cohort |
| Sonderegger *et al.*[96] | 2010 | 3 | 21 | 14.7 | Prospective cohort |
| Stevenson, *et al.*[99] | 2009 | 3 | 18 | 3 | Prospective case series |
| Arora *et al.*[76] | 2007 | 3 | 55 | 14.9 | Retrospective case series |
| Arora *et al.*[77] | 2007 | 3 | 49 | 15.4 | Retrospective case series |
| Strohm *et al.*[100] | 2007 | 3 | 24 | 18 | Retrospective case series |
| Voigt *et al.*[71] | 2006 | 3 | 16 | 9 | Retrospective cohort |
| Jubel *et al.*[55] | 2005 | 3 | 26 | 26 | Retrospective cohort |
| Köck *et al.*[86] | 2005 | 3 | 320 | 12 | Prospective case series |
| Beharrie *et al.*[78] | 2004 | 3 | 3 | 38 | Retrospective cohort |
| Orbay *et al.*[90] | 2004 | 3 | 15 | 16 | Retrospective case series |
| Prokop *et al.*[92] | 2004 | 3 | 15 | 12.1 | Prospective case series |
| Sakhaii *et al.*[93] | 2003 | 3 | 10 | 10 | Prospective case series |
| Schütz *et al.*[94] | 2003 | 3 | 10 | 6 | Prospective case series |
| Zimmerman *et al.*[104] | 1998 | 3 | 30 | 42 | Retrospective case series |
|  |  |  |  |  |  |
| Chilakamary *et al.*[108] | 2016 | 4 | 12 | 9 | Prospective case series |
| Kumaravel *et al.*[33] | 2015 | 4 | 23 | 12 | Prospective cohort |
| Aita *et al.*[9] | 2014 | 4 | 16 | 12 | RCT |
| Kateros *et al.*[111] | 2010 | 4 | 28 | 12 | Retrospective cohort |
| Tyllianakis *et al.*[117] | 2010 | 4 | 8 | 12,5 | Retrospective case series |
| Andersen *et al.*[107] | 2009 | 4 | 75 | 12 | Prospective case series |
| Krukhaug *et al.*[112] | 2009 | 4 | 75 | 12 | RCT |
| Mehboob *et al.*[113] | 2008 | 4 | 30 | 6 | Prospective case series |
| Gradl *et al.*[109] | 2005 | 4 | 14 | 24 | Prospective case series |
| Moroni *et al.*[35] | 2004 | 4 | 20 | 3 | RCT |
| Franck *et al.*[52] | 2000 | 4 | 20 | 6 | RCT |
| Joosten *et al.*[110] | 1999 | 4 | 46 | 28 | Prospective case series |
| Rikli *et al.*[115] | 1998 | 4 | 12 | 63.6 | Retrospective case series |
| Putnam *et al.*[114] | 1997 | 4 | 7 | 14 | Retrospective cohort |
| Horne *et al.*[29] | 1990 | 4 | 15 | N.D. | RCT |
| Schmalholz *et al.*[116] | 1990 | 4 | 27 | 12 | RCT |
|  |  |  |  |  |  |
| Chen *et al.*[118] | 2017 | 5 | 16 | 12 | Retrospective case series |
| Solarino *et al.*[95] | 2016 | 5 | 29 | 12 | Retrospective cohort |
| Plate *et al.*[91] | 2015 | 5 | 30 | 24 | RCT |
| Gradl *et al.*[7] | 2014 | 5 | 66 | 24 | RCT |
| Aita *et al.*[9] | 2014 | 5 | 16 | 12 | RCT |
| Dremstrup *et al.*[120] | 2013 | 5 | 44 | 12 | Prospective cohort |
| Tan *et al.*[42] | 2012 | 5 | 18 | 12 | Retrospective cohort |
| Chen *et al.*[119] | 2012 | 5 | 12 | 11 | Retrospective case series |
| Chappuis *et al.*[80] | 2011 | 5 | 16 | 6 | RCT |
| Takada *et al.*[122] | 2011 | 5 | 14 | 9.5 (3–18) | Retrospective case series |
| Gradl *et al.*[121] | 2009 | 5 | 63 | 12 | Retrospective case series |

Treatments: 1) Plaster cast immobilization, 2) K-wire fixation, 3) Volar plate fixation, 4) External fixator, 5) Intramedullary fixation
