# Supplementary material for: Treatment options in extra-articular distal radius fractures: a systematic review and meta-analysis
Source: Eur J Trauma Emerg Surg. 2021 May 19;48(6):4333–48. doi: 10.1007/s00068-021-01679-z (PMC9712287; doi:10.1007/s00068-021-01679-z)
Supplement: Supplementary file 4 — Supplementary file4 (DOCX 28 kb) [file 68_2021_1679_MOESM4_ESM.docx]

**Supplementary Table 2: Risk of bias assessment per included study**

| **Publication** | **Representativeness  off the exposed cohort** | **Selection of the  non-exposed cohort** | **Ascertainment of  exposure** | **Demonstration that outcome of interest was not present at start of study** | **Comparability of cohorts on  the basis of the design or analysis** | **Assessment of outcome** | **Long enough follow-up for outcomes to occur** | **Adequacy of  follow-up of  cohorts** | **Total stars** |
| --- | --- | --- | --- | --- | --- | --- | --- | --- | --- |
| Gutiérrez-Monclus *et al.[47]* | * | * | * | * | - | * | - | - | 5 |
| Gutiérrez-Espinoza *et al.*[28] | * | * | * | * | ** | * | - | * | 8 |
| Venkatesh *et al.*[44] | * | * | * | * | - | - | - | - | 4 |
| Baruah *et al.*[21] | - | * | * | - | - | * | - | - | 3 |
| Kumaravel *et al.*[33] | - | * | * | * | ** | * | * | - | 7 |
| Subramaniam *et al.*[41] | * | * | * | * | ** | * | * | - | 8 |
| Jellad *et al.*[30] | - | * | * | * | - | * | * | * | 6 |
| Bentohami *et al.*[23] | * | - | * | * | - | * | * | - | 5 |
| Tan *et al.*[42] | * | - | * | * | - | * | * | * | 6 |
| Eastley *et al.*[25] | * | * | * | - | - | * | - | - | 4 |
| Mardani *et al.*[17] | - | * | * | * | ** | * | - | * | 7 |
| Wong, *et al.*[46] | - | * | * | * | ** | * | * | * | 8 |
| Kumar *et al.*[32] | * | * | * | * | - | * | * | * | 7 |
| Rajan *et al.*[36] | - | * | * | - | - | * | - | * | 4 |
| Kulej *et al.*[31] | * | - | * | - | - | * | * | - | 4 |
| Azzopardi *et al.*[20] | - | * | * | * | ** | * | * | * | 8 |
| Moroni *et al.*[35] | - | * | * | - | ** | * | - | - | 5 |
| Leone *et al.*[34] | * | * | * | - | - | * | - | - | 4 |
| Smilovic *et al.*[37] | * | * | * | - | - | * | - | - | 4 |
| Stoffelen *et al.*[40] | - | - | * | * | ** | * | * | - | 6 |
| Basso *et al.*[22] | * | * | * | * | - | * | - | * | 6 |
| Tsukazaki *et al.*[43] | * | * | * | * | - | * | * | - | 6 |
| Field *et al.*[26] | * | - | * | * | - | * | * | - | 5 |
| Gupta *et al.*[27] | * | * | * | - | - | * | * | - | 5 |
| Stein *et al.*[38] | - | * | * | - | * | * | * | * | 6 |
| Horne *et al.*[29] | - | - | * | * | ** | * | - | - | 5 |
| Af Ekenstam *et al.*[19] | * | - | * | - | * | * | * | * | 6 |
| Abbaszadegan *et al.*[18] | * | * | * | - | - | * | * | - | 5 |
| Stewart *et al.*[39] | * | * | * | - | - | * | - | - | 4 |
| Wahlstrom *et al.*[45] | - | - | * | - | - | * | - | * | 3 |
| Blitchert *et al.*[24] | * | * | * | - | - | * | * | * | 6 |
| Camus *et al.[75]* | - | - | * | - | - | * | - | * | 3 |
| Manrique *et al.[73]* | * | * | * | * | ** | * | * | - | 8 |
| Vasudevan *et al.[74]* | * | * | * | - | - | * | * | * | 6 |
| Panthi *et al.*[62] | * | * | * | * | - | * | - | - | 5 |
| Chuang *et al.*[49] | - | * | * | - | - | * | - | * | 4 |
| Costa *et al.*[8] | * | * | * | * | ** | * | * | * | 9 |
| Yang *et al.*[72] | * | * | * | * | - | * | * | - | 6 |
| Maire *et al.*[59] | * | - | * | - | ** | * | * | - | 6 |
| Mirhamidi *et al.*[61] | * | * | * | - | ** | * | - | - | 6 |
| Schneiders *et al.*[67] | - | - | * | - | - | * | * | * | 4 |
| Hull *et al.*[54] | - | - | * | - | - | * | * | * | 4 |
| McFayden *et al.*[60] | * | * | * | * | ** | * | - | * | 7 |
| Das *et al.*[51] | * | - | * | - | - | * | - | * | 4 |
| Huard *et al.*[53] | - | - | * | - | - | * | * | * | 4 |
| Kennedy *et al.*[56] | * | * | * | - | - | * | - | * | 5 |
| Sadighi *et al.*[66] | - | - | * | - | - | * | - | * | 3 |
| Kurup *et al.*[58] | - | - | * | - | - | * | - | * | 3 |
| van Aaken *et al.*[69] | * | - | * | - | - | * | * | * | 5 |
| Vatansever *et al.*[70] | * | - | * | * | - | * | - | * | 5 |
| Szyluk *et al.*[68] | - | - | * | * | - | * | - | * | 4 |
| Voigt *et al.*[71] | * | - | * | - | - | * | - | - | 3 |
| Rosati, *et al.*[63] | * | - | * | - | - | * | * | - | 4 |
| Jubel *et al.*[55] | * | - | * | - | - | * | * | * | 5 |
| Ruschel *et al.*[65] | * | * | * | * | - | * | * | - | 6 |
| Kurup *et al.*[57] | - | - | * | - | - | * | - | * | 3 |
| Rosenthal *et al.*[64] | - | - | * | - | - | * | - | - | 2 |
| Franck *et al.*[52] | * | * | * | * | ** | * | - | - | 7 |
| Brady *et al.*[48] | - | - | * | - | - | * | - | * | 3 |
| Clancey *et al.*[50] | * | - | * | - | - | * | * | * | 5 |
| Shimura *et al.[106]* | - | - | * | - | - | * | * | * | 4 |
| Selles *et al.[105]* | * | - | * | - | - | * | * | * | 5 |
| Thorninger *et al.*[101] | * | - | * | - | - | * | * | * | 5 |
| Zhang *et al.*[103] | * | * | * | * | * | * | * | - | 7 |
| Naito *et al.*[89] | - | - | * | - | - | * | - | * | 3 |
| Solarino *et al.*[95] | * | * | * | * | - | * | * | - | 6 |
| Disseldorp *et al.*[81] | - | * | * | * | - | * | * | - | 5 |
| Plate *et al.*[91] | * | * | * | * | ** | * | * | * | 9 |
| Yamashita *et al.*[102] | - | - | * | * | * | * | - | * | 5 |
| Huffaker *et al.*[85] | - | - | * | * | - | * | - | * | 4 |
| Häberle *et al.*[84] | * | * | * | - | * | * | - | * | 6 |
| Gereli *et al.*[82] | - | * | * | * | * | * | * | * | 7 |
| Gradl *et al.*[7] | * | * | * | - | ** | * | * | - | 7 |
| Lebailly *et al.*[87] | * | - | * | - | - | * | - | * | 4 |
| Aita *et al.*[9] | * | * | * | * | ** | * | * | - | 8 |
| Mignemi *et al.*[88] | - | - | * | * | - | * | - | * | 4 |
| Braziulis *et al.*[79] | - | - | * | - | - | * | - | * | 3 |
| Chappuis *et al.*[80] | * | * | * | - | ** | * | - | * | 7 |
| Geyer *et al.*[83] | * | - | * | * | - | * | - | * | 5 |
| Souer, *et al.*[97] | - | - | * | * | * | * | * | * | 6 |
| Souer, *et al.*[98] | - | * | * | * | * | * | * | - | 6 |
| Sonderegger *et al.*[96] | - | - | * | - | - | * | * | * | 4 |
| Stevenson, *et al.*[99] | - | - | * | - | - | * | - | - | 3 |
| Arora *et al.*[76] | - | - | * | * | - | * | * | - | 4 |
| Arora *et al.*[77] | * | - | * | - | - | * | * | - | 4 |
| Strohm *et al.*[100] | * | - | * | - | - | * | * | - | 4 |
| Köck *et al.*[86] | - | - | * | - | - | * | * | - | 3 |
| Beharrie *et al.*[78] | * | - | * | - | - | * | * | * | 5 |
| Orbay *et al.*[90] | - | - | * | - | - | * | * | * | 4 |
| Prokop *et al.*[92] | * | - | * | - | - | * | * | * | 5 |
| Sakhaii *et al.*[93] | - | - | * | - | - | * | - | * | 3 |
| Schütz *et al.*[94] | * | - | * | - | - | * | - | * | 4 |
| Zimmerman *et al.*[104] | * | - | * | - | - | * | * | * | 5 |
| Chilakamary *et al.*[108] | * | - | * | * | - | * | - | * | 5 |
| Kateros *et al.*[111] | * | * | * | * | - | * | * | - | 6 |
| Tyllianakis *et al.*[117] | - | * | * | - | - | * | * | * | 5 |
| Andersen *et al.*[107] | * | * | * | - | - | * | * | - | 5 |
| Krukhaug *et al.*[112] | * | * | * | * | * | * | * | * | 8 |
| Mehboob *et al.*[113] | - | - | * | - | - | * | - | - | 2 |
| Gradl *et al.*[109] | * | - | * | * | - | * | * | * | 5 |
| Joosten *et al.*[110] | * | - | * | - | - | * | * | - | 4 |
| Rikli *et al.*[115] | - | - | * | - | - | * | * | - | 3 |
| Putnam *et al.*[114] | - | - | * | - | - | * | - | * | 3 |
| Schmalholz *et al.*[116] | - | * | * | * | ** | * | * | * | 8 |
| Chen *et al.*[118] | * | - | * | - | - | * | * | * | 5 |
| Dremstrup *et al.*[120] | * | * | * | - | - | * | * | - | 5 |
| Chen *et al.*[119] | - | - | * | - | - | * | - | * | 3 |
| Takada *et al.*[122] | * | - | * | - | - | * | * | - | 4 |
| Gradl *et al.*[121] | * | - | * | - | - | * | * | * | 5 |

a. Awarded one star if cohort included all extra-articular fractures and did not restrict cohort.

b. Awarded one star as comorbidities and pre-existent function restrictions were excluded.

c. Awarded one star if there was certainty of presence of extra-articular distal radius fracture.

d. Awarded one star if it was clearly stated whether it concerned fresh fractures (<14days) and non-recurrent fractures

e. Awarded one star if study design adjusted for baseline characteristics (i.e. Age, female-male ratio, osteoporosis, time to presentation at ER). Additional star awarded to any additional adjustments.

f. Awarded one star if outcome is assessed objectively (i.e. x-rays, medical records, etc)

g. One star awarded if outcome assessed until one year after trauma/intervention

h. One star awarded if loss to follow-up was less than 10%
