# Supplementary material for: Treatment options in extra-articular distal radius fractures: a systematic review and meta-analysis
Source: Eur J Trauma Emerg Surg. 2021 May 19;48(6):4333–48. doi: 10.1007/s00068-021-01679-z (PMC9712287; doi:10.1007/s00068-021-01679-z)
Supplement: Supplementary file 5 — Supplementary file5 (DOCX 24 kb) [file 68_2021_1679_MOESM5_ESM.docx]

**Supplementary Table 3: Pooled radiographic outcomes per treatment**

| **Parameter** | **Treatment** | **Pooled estimate 3mo** | **Pooled estimate 6mo** | **Pooled estimate 12mo** | **Pooled estimate >12mo** |
| --- | --- | --- | --- | --- | --- |
| Volar tilt | Nonoperative | -5.2 [8;535] | -3.0 [1;30] | -3.4 [3;67] | -3.2 [2;279] |
|  | K-wire | 2.2 [15;601] | 7.8 [7;259] | -0.2 [8;213] | 7.0 [3;57] |
|  | Volar plate | 8.5 [6;263] | 6.5 [5;210] | 5.5 [7;620] | 4.2 [12;420] |
|  | External fixator | 6.6 [3;170] | 6.7 [1;20] | 5.2 [3;178] | 7.1 [3;29] |
|  | IMN | 3.0 [4;88] | -2.1 [1;16] | 5.1 [4;103] | 5.3 [2;96] |
| Radial inclination | Nonoperative | 15.8 [5;358] | 16.0 [1;30] | 17.4 [2;57] | 22.6 [2;279] |
|  | K-wire | 21.0 [11;450] | 20.7 [4;95] | 21.3 [6;193] | 19.3 [3;57] |
|  | Volar plate | 22.7 [6;263] | 22.7 [5;210] | 25.3 [7;620] | 22.4 [11;388] |
|  | External fixator | 23.7 [3;170] | 24.1 [1;20] | 23.1 [3;178] | 22.8 [2;22] |
|  | IMN | 21.6 [4;88] | 23.4 [1;16] | 20.5 [4;103] | 22.0 [1;30] |
| Radial Height | Nonoperative | 4.5 [4;267] | 4.2 [1;30] | 4.6 [2;57] | 9.8 [1;22] |
|  | K-wire | 10.2 [10;396] | 10.5 [4;107] | 9.6 [5;178] | 10.1 [1;29] |
|  | Volar plate | 11.3 [3;88] | 11.0 [1;10] | 11.0 [1;31] | 11.1 [3;87] |
|  | External fixator | 12.0 [1;75] | NA. | 10 [1;28] | NA. |
|  | IMN | 11.6 [3;72] | NA. | 11.1 [2;60] | 10.0 [1;30] |
| Ulnar Variance | Nonoperative | 2.1 [6;463] | 3.2 [1;30] | 2.9 [3;67] | 0.5 [2;279] |
|  | K-wire | 2.0 [10;471] | 1.4 [5;185] | 2.3 [6;187] | 0.6 [3;57] |
|  | Volar plate | 0.3 [5;201] | -0.1 [4;200] | 0.3 [6;558] | 0.3 [11;381] |
|  | External fixator | 0.7 [2;95] | 0.8 [1;20] | 0.5 [1;75] | 0.2 [3;29] |
|  | IMN | 1.2 [1;16] | 2.0 [1;16] | 0.0 [2;43] | 0.38 [2;96] |

Data are shown as pooled estimate [N studies; N patients].
