# Supplementary material for: Treatment options in extra-articular distal radius fractures: a systematic review and meta-analysis
Source: Eur J Trauma Emerg Surg. 2021 May 19;48(6):4333–48. doi: 10.1007/s00068-021-01679-z (PMC9712287; doi:10.1007/s00068-021-01679-z)
Supplement: Supplementary file 6 — Supplementary file6 (DOCX 23 kb) [file 68_2021_1679_MOESM6_ESM.docx]

**Supplementary Table 4: Pooled consolidation rate and Lidstrom Score at >12 months follow-up**

| **Parameter** | **Treatment** | **Studies**  **(N)** | **Population**  **(N)** | **Q**  **(p-value)** | **I^2^**  **(95% CI)** | **Pooled estimate**  **(95% CI)** |
| --- | --- | --- | --- | --- | --- | --- |
| Consolidation | Nonoperative | 5 | 263 | 0.3 (0.987) | 0 (0-0) | 99.6 (97.8-100) |
|  | K-wire | 11 | 451 | 1.0 (1.000) | 0 (0-0) | 99.5 (98.3-100) |
|  | Volar plate | 8 | 346 | 13.3 (0.065) | 47.3 (0-76.6) | 99.5 (98.1-99.9) |
|  | External fixator | 2 | 50 | 0.02 (0.889) | 0 (0-0) | 99.0 (91.4-100) |
|  | IMN | 3 | 44 | 0.02 (0.990) | 0 (0-0) | 98.4 (89.6-100) |
| Lidstrom score Good/Excellent | Nonoperative | 3 | 143 | 21.4 (<0.001) | 90.7 (75.5-96.4) | 71.9 (45.3-92.2) |
|  | K-wire | 2 | 84 | 0.52 (0.470) | 0 (0-0) | 89.7 (81.3-95.2) |
|  | Volar plate | NA. | NA. | NA. | NA. | NA. |
|  | External fixator | 3 | 97 | 21.7 (<0.001) | 90.8 (75.9-96.5) | 87.9 (59.9-99.9) |
|  | IMN | NA. | NA. | NA. | NA. | NA. |
