# Supplementary material for: Treatment options in extra-articular distal radius fractures: a systematic review and meta-analysis
Source: Eur J Trauma Emerg Surg. 2021 May 19;48(6):4333–48. doi: 10.1007/s00068-021-01679-z (PMC9712287; doi:10.1007/s00068-021-01679-z)
Supplement: Supplementary file 7 — Supplementary file7 (DOCX 24 kb) [file 68_2021_1679_MOESM7_ESM.docx]

**Supplementary Table 5: Pooled range of motion and grip strength per treatment**

| **Parameter** | **Treatment** | **Pooled estimate 3mo** | **Pooled estimate 6mo** | **Pooled estimate 12mo** | **Pooled estimate >12mo** |
| --- | --- | --- | --- | --- | --- |
| Flexion (degrees) | Nonoperative | 55.2 [1;74] | 62.5 [2;104] | 63.5 [1;10] | 66.1 [2;52] |
|  | K-wire | 42.0 [4;135] | 60.9 [3;79] | 56.2 [3;74] | 63.4 [3;59] |
|  | Volar plate | 65.4 [4;176] | 56.1 [4;179] | 58.4 [6;609] | 59.5 [11;433] |
|  | External fixator | 57.0 [1;20] | 68.6 [2;50] | 60.0 [1;28] | 68.2 [1;7] |
|  | IMN | NA. | 62.5 [1;16] | 62.7 [2;30] | NA. |
| Extension  (degrees) | Nonoperative | 60.9 [1;74] | 65.6 [2;104] | 64.9 [1;10] | 65.9 [2;52] |
|  | K-wire | 42.9 [4;135] | 60.8 [3;79] | 59.1 [3;74] | 61.5 [3;59] |
|  | Volar plate | 59.0 [4;176] | 59.9 [4;179] | 60.8 [6;609] | 62.1 [11;433] |
|  | External fixator | 66.0 [1;20] | 79.0 [2;50] | 58.0 [1;28] | 66.4 [1;7] |
|  | IMN | NA. | 42.5 [1;16] | 65.7 [2;30] | NA. |
| Ulnar deviation  (degrees) | Nonoperative | NA. | 22.0 [1;30] | NA. | 27.8 [2;52] |
|  | K-wire | 27.5 [3;99] | 26.1 [3;79] | 21.9 [1;29] | 24.8 [2;44] |
|  | Volar plate | 18 [1;31] | 30.7 [4;179] | 36.2 [6;499] | 31.0 [6;301] |
|  | External fixator | 34.1 [2;34] | 44.2 [2;50] | 25.0 [1;28] | 21.0 [1;14] |
|  | IMN | NA. | NA. | 23.5 [1;29] | NA. |
| Radial deviation (degrees) | Nonoperative | NA. | 16 [1;30] | NA. | 24.4 [2;52] |
|  | K-wire | 15.4 [3;99] | 19.6 [3;79] | 15.7 [1;29] | 20.7 [2;44] |
|  | Volar plate | 6.1 [1;31] | 19.6 [4;179] | 23.6 [6;499] | 21.5 [6;301] |
|  | External fixator | 22.9 [2;34] | 17.2 [2;50] | 18.0 [1;28] | 23.0 [1;14] |
|  | IMN | NA. | NA. | 10.3 [1;29] | NA. |
| Pronation (degrees) | Nonoperative | NA. | 61.0 [1;30] | NA. | 77.5 [2;52] |
|  | K-wire | 66.6 [2;79] | 67.3 [2;59] | 71.4 [1;29] | 81.1 [2;44] |
|  | Volar plate | 81.4 [3;145] | 82.1 [4;179] | 88.3 [6;588] | 81.8 [9;420] |
|  | External fixator | 74.0 [1;14] | NA. | 71.0 [1;28] | 81.5 [2;21] |
|  | IMN | NA. | 85.0 [1;16] | 81.9 [1;16] | NA. |
| Supination (degrees) | Nonoperative | NA. | 64.0 [1;30] | NA. | 78.8 [2;52] |
|  | K-wire | 59.9 [2;79] | 64.3 [2;59] | 66.6 [1;29] | 78.9 [2;44] |
|  | Volar plate | 80.0 [4;175] | 80.2 [4;179] | 85.3 [6;588] | 82.1 [10;423] |
|  | External fixator | 71.0 [1;14] | NA. | 72.0 [1;28] | 79.8 [2;21] |
|  | IMN | 87.0 [1;30] | 80.0 [1;16] | 76.6 [1;16] | NA. |
| Grip strength (%) | Nonoperative | 54.0 [2;93] | 69.1 [2;93] | 75.3 [3;56] | 96.0 [1;58] |
|  | K-wire | 58.8 [2;56] | 94.0 [1;20] | 79.6 [2;36] | 80.3 [4;74] |
|  | Volar plate | 68.5 [5;191] | 70.1 [1;83] | 87.7 [2;122] | 89.8 [8;264] |
|  | External fixator | 47.9 [2;39] | 85.0 [1;20] | 94.5 [1;16] | 93.0 [1;12] |
|  | IMN | 49.4 [4;127] | 80.5 [2;81] | 89.9 [4;111] | 90.2 [2;96] |

Data are shown as pooled estimate [N studies; N patients].
