# Supplementary material for: Treatment options in extra-articular distal radius fractures: a systematic review and meta-analysis
Source: Eur J Trauma Emerg Surg. 2021 May 19;48(6):4333–48. doi: 10.1007/s00068-021-01679-z (PMC9712287; doi:10.1007/s00068-021-01679-z)
Supplement: Supplementary file 8 — Supplementary file8 (DOCX 24 kb) [file 68_2021_1679_MOESM8_ESM.docx]

**Supplementary Table 6: Pooled functional pain and outcome scores per treatment**

| **Parameter** | **Treatment** | **Pooled estimate 3mo** | **Pooled estimate 6mo** | **Pooled estimate 12mo** | **Pooled estimate >12mo** |
| --- | --- | --- | --- | --- | --- |
| DASH | Nonoperative | 67.1 [2;199] | 31.6 [2;199] | 8.0 [1;19] | NA. |
|  | K-wire | 34.3 [2;65] | 23.4 [2;65] | 14.8 [2;88] | 17.9 [4;98] |
|  | Volar plate | 13.0 [4;180] | 16.7 [4;101] | 7.6 [8;337] | 8.0 [7;341] |
|  | External fixator | 29.5 [1;16] | NA. | 9.4 [2;91] | NA. |
|  | IMN | 15.0 [2;34] | 16.1 [2;34] | 14.3 [3;63] | NA. |
| Gartland & Werley % Good/Excellent | Nonoperative | 51.7 [1;29] | 81.9 [2;94] | NA. | 89.7 [1;29] |
|  | K-wire | 42.2 [2;45] | 84.1 [4;107] | 94.2 [1;52] | 87.9 [3;58] |
|  | Volar plate | NA. | NA. | 87.5 [1;16] | 79.3 [2;29] |
|  | External fixator | NA. | NA. | 81.6 [2;103] | 66.7 [2;54] |
|  | IMN | NA. | NA. | 93.2 [1;44] | NA. |
| Gartland & Werley score | Nonoperative | NA. | 5.2 [1;30] | 14.3 [1;25] | NA. |
|  | K-wire | 14.7 [4;110] | 11.7 [4;104] | NA. | 3.7 [3;81] |
|  | Volar plate | 9.6 [2;58] | 6.0 [3;163] | 3.3 [3;153] | 2.5 [6;263] |
|  | External fixator | NA. | NA. | 3.9 [1;23] | 1.3 [2;21] |
|  | IMN | 6.0 [1;63] | 1.7 [1;63] | 4.7 [2;92] | 2.7 [1;66] |
| VAS | Nonoperative | 2.2 [1;74] | 1.4 [1;74] | 1.2 [1;27] | NA. |
|  | K-wire | NA. | NA. | 0.7 [1;27] | 2.3 [1;29] |
|  | Volar plate | 1.4 [2;24] | 1.5 [3;219] | 1.0 [4;169] | 1.0 [4;217] |
|  | External fixator | 1.3 [2;30] | NA. | 0.7 [2;91] | 0.2 [1;14] |
|  | IMN | 2.4 [2;79] | 1.4 [1;63] | 0.7 [5;168] | 1.0 [1;66] |

Data are shown as pooled estimate [N studies; N patients].
